# On Fixed-Wing Drone-Enabled Covert Transmission with Mobility Restrictions and QoS Requirement

**DOI:** 10.3390/s26103159

**Published:** 2026-05-16

**Authors:** Cheng He, Xiaodong Ji, Lili Guo

**Affiliations:** 1 School of Information Science and Technology, Nantong University, Nantong 226019, China; hecheng@stmail.ntu.edu.cn (C.H.); xlguolili@ntu.edu.cn (L.G.); 2 Xinglin College, Nantong University, Nantong 226236, China

**Keywords:** fixed-wing drone, covert rate, mobility restriction, QoS requirement

## Abstract

**Highlights:**

**What are the main findings?**

- A joint optimization algorithm is developed to maximize the covert rate by adjusting a fixed-wing drone’s transmit power and speed, and seeking the optimal central position and radius of its flight area.
- To maximize the covert rate, there exists a distance threshold between the receiver and the detector that is determined by the drone’s minimum flight speed and maximum bank angle. Below the threshold, the drone should fly as high as possible; above it, as low as possible.

**What are the implications of the main findings?**

- The proposed optimization algorithm can fully utilize the fixed-wing drone’s maneuverability to select both the flight area and altitude based on the distance from the detector.
- Within the derived flight area, the drone is capable of delivering covert data at the optimized covert rate while meeting the required level of quality of service.

**Abstract:**

This article examines a fixed-wing drone (FWD)-enabled covert transmission, where the FWD performs a level flight within a circular area with a radius of $r_{R}$ meters and serves as a mobile transmitter to covertly deliver data to a ground receiver situated $d_{0}$ meters away from a ground detector. First, the conditions for satisfying the covertness constraint and meeting the transmission’s quality of service (QoS) requirement are derived, imposing additional mobility restrictions on the FWD beyond the predefined speed and altitude limits. Considering the FWD’s mobility and transmit power limits, an optimization problem maximizing the covert rate is solved, leading to a joint optimization algorithm that adjusts the FWD’s transmit power and speed while seeking the optimal central position and radius of the circular area. Within this area, the FWD is capable of performing an unrestricted level flight and delivering covert data at the optimized covert rate while meeting the required QoS level. Computer simulation results demonstrate that the smaller the radius $r_{R}$, the larger the covert rate will be. The minimum value of $r_{R}$ is twice the FWD’s minimum turning radius, which is determined by the minimum flight speed and the maximum bank angle. The FWD’s flight altitude exhibits a strong correlation with both $r_{R}$ and $d_{0}$. If $2r_{R}<d_{0}\leq 4r_{R}$, the FWD should fly as high as possible. Conversely, if $d_{0}>4r_{R}$, the FWD should fly as low as possible so as to maximize the covert rate.

## 1. Introduction

Recently, drones, relying on their controllable high mobility, flexible on-demand deployment, and high probability of establishing line-of-sight (LoS) connections, have provided new avenues for supplementing wireless services while improving transmission range and quality in specific environments [1]. In practical applications, two types of drones (i.e., rotor-wing and fixed-wing drones) with necessary communication facilities can be deployed to offer data delivery or relaying services for air and ground users [2]. Compared with rotary-wing drones (RWDs), fixed-wing drones (FWDs) have inherent merits in various aspects, such as long endurance and large payloads, and hence play an increasingly important role in civilian, military, and commercial applications [2]. However, FWDs cannot hover in the air and must always fly forward so as to obtain lift [3]. Thus, FWDs face greater mobility restrictions and inferior maneuverability compared to RWDs. This flight characteristic results in two constraints for covert communications. First, FWDs cannot hover and must always fly forward to stay in the air. Second, unlike rotor-wing drones (RWDs), which can hover in place and change position freely, FWDs have a turning radius constrained by speed and bank angle, preventing arbitrarily sharp turns. Consequently, most covert communication schemes designed for RWDs are not suitable for FWDs.

As mentioned earlier, drone-assisted air-to-ground and ground-to-air transmissions are most likely to benefit from LoS connections, resulting in not only an improvement in transmission quality but also an increase in the risk of being detected or intercepted [2]. Covert communication, also referred to as low probability of detection communication, is expected to solve privacy protection and security issues in wireless communications [4]. The fundamental idea of covert communication is to handle data delivery from a transmitter to a receiver, subject to a constraint on a detector’s detection error probability (DEP). If the DEP is kept above 1−ϑ for a positive and arbitrarily small ϑ, the communication behavior is presumed to be hidden from the detector [5]. Nowadays, to protect communication security, the idea of covert communication has been implemented in many fields such as underwater acoustic communication [6], the internet of vehicles [7] and satellite–terrestrial networks [8].

In view of their merits, drones are also capable of enhancing the covertness of wireless transmissions; hence, drone-enabled covert technologies have recently attracted much attention. In [9,10,11,12,13], drones are deployed to serve as legitimate transmitters or receivers to deliver or collect data. Then, transmit power control and optimal placement or trajectory are examined so as to optimize the transmission quality while satisfying the covertness constraint. Provided that the drone is a flying detector or a friendly jammer, effective strategies are devised so that data deliveries between ground transceivers are hidden [14,15]. By combining with advanced communication techniques, such as non-orthogonal multiple access, intelligent reflecting surface, maximum ratio transmission and zero-forcing, drone-enabled covert transmissions are examined in [16,17,18,19], leading to various types of covert communication technologies. Considering specific wireless environments such as cognitive radio, and integrated sensing and communication, drones are employed to hide target transmissions from intended detectors [20,21].

It should be noted that in the aforementioned studies, the drones are assumed to either remain stationary or follow fixed trajectories (from a start to an end location) with constrained speeds and altitudes. This scenario essentially models an RWD-enabled covert transmission. Given their inherent merits, FWDs are widely deployed to assist wireless transmissions, especially in the military area, where communication security is of utmost importance [2]. Therefore, it is imperative to examine FWD-enabled covert transmissions. As mentioned earlier, FWDs face greater mobility restrictions than RWDs and their maneuverability is not only affected by flight speed and altitude but also by factors such as the maximum bank angle [3]. Therefore, for FWD-enabled covert transmissions, not only DEP constraint but also the drone’s mobility restrictions should be considered. To the authors’ best knowledge, such an issue is still open and requires further research.

This article considers an FWD-enabled covert transmission, where the drone performs a level flight within a circular area with a radius of $r_{R}$ meters and serves as a mobile transmitter to covertly deliver data to Bob situated $d_{0}$ meters away from Willie. With the aim of maximizing the covert rate, a joint optimization problem is established and solved, leading to a new optimization algorithm. The main contributions of this article are summarized as follows.

- Provided that Willie is equipped with a power detector so as to identify potential communication behaviors, the conditions for satisfying the covertness constraint and meeting the transmission’s quality of service (QoS) requirement are derived, imposing additional mobility restrictions on the FWD beyond the predefined speed and altitude limits.
- Considering the FWD’s mobility and transmit power limits, an optimization problem maximizing the covert rate is solved, leading to a joint optimization algorithm that adjusts the FWD’s transmit power and speed while seeking the optimal central position and radius of the circular area. Within this area, the FWD is capable of performing an unrestricted level flight and delivering covert data at the optimized covert rate while meeting the required QoS level.
- Computer simulations are conducted to verify the correctness and effectiveness of the investigations. It demonstrates that the smaller the radius $r_{R}$, the larger the covert rate will be. The minimum value of $r_{R}$ is twice the FWD’s minimum turning radius, which is determined by the minimum flight speed and the maximum bank angle. The FWD’s flight altitude is strongly correlated with the values of $r_{R}$ and $d_{0}$. If $2r_{R}<d_{0}\leq 4r_{R}$, the FWD should fly as high as possible. Conversely, if $d_{0}>4r_{R}$, the FWD should fly as low as possible so as to maximize the covert rate.

The remainder of this article is organized as follows. The examined system model is presented in Section 2. In Section 3, the conditions for satisfying the covertness constraint and meeting the transmission’s QoS requirement are derived. On this basis, an optimization problem maximizing the covert rate is established. In Section 4, the optimization problem is solved, leading to a joint optimization algorithm that adjusts the FWD’s transmission power and speed while seeking the optimal central position and radius of the circular area. Simulation results and discussions are presented in Section 5 so as to verify the correctness and effectiveness of the proposed optimization algorithm. Section 6 concludes this article.

## 2. Related Work

Research on drone-enabled covert communications have grown considerably in recent years. A comprehensive survey by Yang et al. outlines the current research status and key technologies of covert drone communications [2]. Early work by Zhou et al. first attempted to jointly optimize the trajectory and transmit power of a single drone to maximize the average covert rate [22]. Jiang et al. extended this approach to multi-user scenarios in [10], and Wu et al. further demonstrated that the optimal trajectory followed a successive-hover-and-fly structure, which reduced complexity [11]. For scenarios involving multiple wardens, Lei et al. introduced a jamming drone and jointly optimized the trajectories and transmit powers of both drones to maximize the minimum average covert rate among ground users [23]. However, all these works assume that the drone can hover or change direction freely. While this assumption holds for RWDs, it is not valid for FWDs, which cannot hover and, due to their mobility constraints, must maintain a minimum turning radius.

Beyond single-drone systems, research has also explored multi-drone and relay-assisted schemes. Yang et al. optimized the altitude and power of multiple drone relays with relay selection to mitigate small-scale fading in dense urban environments [24]. Liu et al. studied dual-drone short-packet covert communications under warden location uncertainty, jointly optimizing 3D trajectories and power [25]. To cope with active wardens, several jamming-assisted strategies have emerged. Wei et al. employed fluid antenna arrays to actively counter active wardens [26]. The authors of [27] proposed a cognitive jamming protocol, where a friendly jammer transmitted only when the legitimate transmitter was idle. The authors of [28] investigated robust covert communication against active wardens under location uncertainty. Zhang et al. examined drone relay-assisted cooperative jamming over mixed fading channels and derived the optimal power allocation [29]. Reconfigurable intelligent surfaces (RISs) have also been explored in this context. The authors of [30] used a STAR-RIS to assist non-orthogonal multiple access transmission, while the authors of [31] mounted an IRS on a drone as a mobile relay, jointly optimizing the trajectory and phase shifts. Emerging techniques such as deep reinforcement learning have also been applied. The authors of [32] applied it to minimize the peak age of information of public Internet of Things devices under covertness constraints. Note that most of the existing works implicitly assume RWDs. In addition to the studies mentioned above, Zeng et al. minimized the energy consumption of an RWD by leveraging its hovering capability [33]. This fundamentally differs FWDs, which cannot hover and are subject to a minimum turning radius. To the authors’ best knowledge, none of the above studies explicitly account for the fact that FWDs cannot hover and must satisfy a minimum turning radius. This article precisely fills this gap.

## 3. System Model

**Figure 1:**
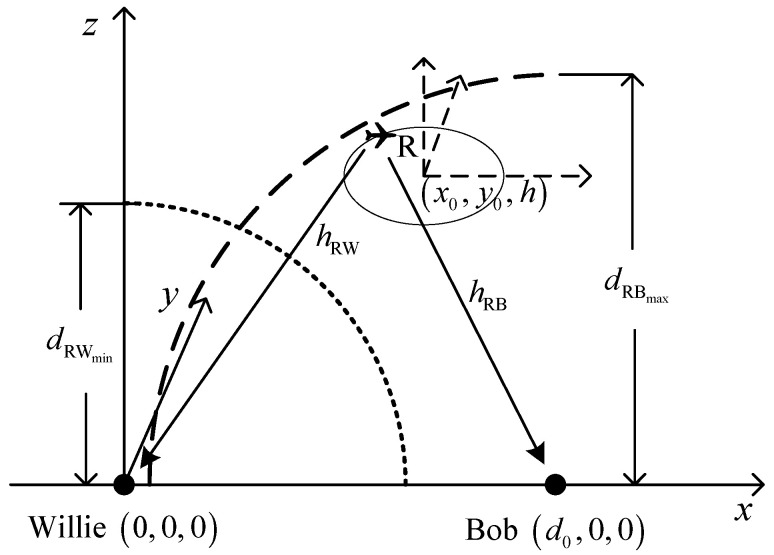
System model.

Figure 1 shows the examined system model, where an FWD R serves as a mobile transmitter to covertly deliver data to Bob; meanwhile, Willie dedicates to detecting whether such a communication behavior exists. Suppose that R performs an unrestricted level flight within a circular area D, where R is capable of delivering covert data to Bob at any time. Here, that means the FWD operates within a cylindrical region defined by a horizontal disk D of radius $r_{R}$ and an altitude range of $\left[h_{\min},h_{\max}\right]$. Within this cylinder, the UAV can maneuver freely (i.e., follow a spiral trajectory) as long as it respects its kinematic constraints. In addition, the unrestricted level flight means that R can start its flight from the central position of D in any direction and then fly back to the starting position, and during this process, R is always within D without changing its altitude and speed. Denote by T the period of time during which R performs such a flight at a speed of v. Denote by $\left(x_{0},y_{0},h\right)$ and $r_{R}$ the central position and the radius of D, respectively, where $\left(x_{0},y_{0}\right)$ is its horizontal coordinate and h is the FWD’s flight altitude. It should be noted that $\left(x_{0},y_{0},h\right)$ determines the location of D, while $r_{R}$ affects the size of D. Suppose that R only flies in the airspace above Willie and Bob and hence, the location and the radius of D will be restricted, which leads to $r_{R}\leq x_{0}\leq d_{0}-r_{R}$ and $0<r_{R}\leq d_{0}/2$. Willie and Bob are situated at $q_{w}=\left(0,0,0\right)$ and $q_{B}=\left(d_{0},0,0\right)$, respectively, where $d_{0}=\left\|q_{w}-q_{B}\right\|$ is the distance between Willie and Bob, and $q_{w}$ and $q_{B}$ are assumed to be known to R.

As is well known, an FWD must always fly forward so as to obtain lift and hence, there should be a minimum speed limit (also known as stall speed) [3]. On the other hand, an FWD’ s structure will be stressed beyond the limit load factor when it flies in excess of a design maneuvering speed [3]. Such a design maneuvering speed can be considered as a maximum speed limit. Therefore, R’s flight speed is subject to the following constraint.

(1) $$V_{\min}\leq v\leq V_{\max}$$

In Equation (1), $V_{\min}$ and $V_{\max}$ are the minimum and maximum flight speed limits.

According to the 3GPP (3rd Generation Partnership Project), the probability of existing LoS links between air and ground transceivers is 100% when flying at an altitude greater than 40 m above the ground in rural environments [34]. On the other hand, there should be a maximum flight altitude limit due to the consideration of flight safety and/or the local regulations. Therefore, to ensure the existence of LoS links and flight safety, the flight altitude of the FWD should meet

(2) $$h_{\min}\leq h\leq h_{\max},$$

where $h_{\min}$ and $h_{\max}$ are the minimum and maximum altitude limits of the FWD.

In the article, T is divided into N time-slots, and each has an equal length of $T_{N}=T/N\;$. Suppose that $T_{N}$ is sufficiently small; hence, R remains stationary within a time-slot. In the article, $H_{1}$ and $H_{0}$ are used to denote the events in which covert data are and are not delivered, respectively. Suppose that Willie utilizes a power detector to identify such potential communication behavior. Specifically, Willie conducts m numbers of detection in each time-slot, and then computes the detected average power used as the criterion to judge whether the communication behavior occurs. In time-slot n, the i-th detected signal by Willie can be written as [10]

(3) $$y_{W}^{i}\left[n\right]=\left\{\begin{array}{l} n_{W}\left[i\right],H_{0}\\ \sqrt{P_{R}\left[n\right]}h_{\mathrm{RW}}x_{R}^{i}\left[n\right]+n_{W}\left[i\right],H_{1} \end{array}\right.,$$

where $i\in \left\{1,\cdots ,m\right\}$ and $n_{W}\left[i\right]$ denote the noise at Willie’s location with a power of $\delta_{W}$; $x_{R}^{i}\left[n\right]$ denotes the i-th detection of $x_{R}\left[n\right]$, which is a unit power signal and covertly sent by R in time-slot n; $P_{R}\left[n\right]$ is the transmit power of R in the case that $x_{R}\left[n\right]$ is sent in time-slot n and $h_{\mathrm{RW}}$ denotes the channel gain from R to Willie. Since the free space path loss model [11,18] is assumed, $h_{\mathrm{RW}}$ can be written as $h_{\mathrm{RW}}=\sqrt{\beta_{0}/d_{\mathrm{RW}}^{2}}$, where $\beta_{0}$ denotes the channel power at unit distance and $d_{\mathrm{RW}}$ is the distance between R and Willie. It should be noted that $P_{R}\left[n\right]$ is subject to the following constraint

(4) $$P_{\min}\leq P_{R}\left[n\right]\leq P_{\max},$$

where $P_{\min}$ and $P_{\max}$ are the minimum and maximum transmit power limits.

According to the detected signal given by Equation (3), Willie will make a binary judgment $D_{1}$ or $D_{0}$. Here, $D_{1}$ corresponds to the finding of the communication behavior, while $D_{0}$ corresponds to the case that no communication behavior is identified. Similar to [10], the decision rule adopted by Willie can be expressed as

(5) $$T_{W}\left[n\right]≜\frac{1}{m}\sum_{i=1}^{m}{\left|y_{W}^{i}\left[n\right]\right|}^{2}\begin{array}{c} {>}^{D_{1}}\\ D_{0}\leq \end{array}\lambda \left[n\right],$$

where $T_{W}\left[n\right]$ is the average power detected by Willie in time-slot n, ${\left|y_{W}^{i}\left[n\right]\right|}^{2}$ is the signal power detected by Willie during the i-th detection, m is the total number of detections in each time-slot, and $\lambda \left[n\right]$ is the set detection threshold. Suppose that Willie conducts an infinite number of detections, namely, m→∞. Then, $T_{W}\left[n\right]$ can be rewritten as [10,12]

(6) $$T_{W}\left[n\right]=\left\{\begin{array}{l} \delta_{W},H_{0}\\ {\left|h_{\mathrm{RW}}\right|}^{2}P_{R}\left[n\right]+\delta_{W},H_{1} \end{array}\right..$$

In the article, noise uncertainty is considered. In this situation, noise power $\delta_{W}$ becomes a random variable with a known distribution. According to [5,35], the noise power $\delta_{W}$ in the dB domain can be considered as a uniformly distributed random variable within the interval of $\left[\overset{⌣}{\delta }-\rho ,\overset{⌣}{\delta }+\rho \right]$, where $\overset{⌣}{\delta }$ is the mean value in the dB domain and ρ is the noise uncertainty parameter in the dB domain and meets ρ>0. Therefore, the probability density function (PDF) of $\delta_{W}$ can be expressed as [5,35]

(7) $$f_{\delta_{W}}\left(x\right)=\left\{\begin{array}{l} \frac{5}{\ln\left(10\right)}\cdot \frac{1}{\rho x},10^{\frac{\overset{⌣}{\delta }-\rho }{10}}\leq x\leq 10^{\frac{\overset{⌣}{\delta }+\rho }{10}}\\ 0,\mathrm{others} \end{array}\right..$$

Due to the uncertainty about noise power, Willie may make two types of mistakes during the detection process. The first one is called a missed detection, namely, judgment $D_{0}$ is made while $H_{1}$ happens. The second one is called a false alarm, namely, judgment $D_{1}$ is made while $H_{0}$ occurs in practice. Denote by $P_{F}\left[n\right]$ and $P_{M}\left[n\right]$ the probabilities of a false alarm and missed detection occurring, respectively. According to the decision rule given by Equation (5), $P_{F}\left[n\right]$ and $P_{M}\left[n\right]$ can be written as

(8) $$P_{F}\left[n\right]≜\mathrm{Pr}\left\{D_{1}|H_{0}\right\}=\mathrm{Pr}\left\{\delta_{W}>\lambda \left[n\right]\right\},$$

(9) $$P_{M}\left[n\right]≜\mathrm{Pr}\left\{D_{0}|H_{1}\right\}=\mathrm{Pr}\left\{{\left|h_{\mathrm{RW}}\right|}^{2}P_{R}\left[n\right]+\delta_{W}\leq \lambda \left[n\right]\right\}.$$

Then, the DEP is defined as the summation of the false alarm and missed detection probabilities and can be expressed as

(10) $$\varepsilon \left[n\right]=P_{F}\left[n\right]+P_{M}\left[n\right].$$

## 4. Performance Analysis and Problem Establishment

It is worth mentioning that from Willie’s point of view, the power detection should be conducted as precisely as possible, meaning that the DEP given by Equation (10) should be as small as possible. From the FWD’s and Bob’s point of view, however, their goal is to keep their communication behavior undetected by Willie and guarantee their transmission quality as well. To this end, the DEP given by Equation (10) is first calculated, obtaining the minimum DEP $\varepsilon_{\min}\left[n\right]$. On this basis, the condition for satisfying the covertness constraint is derived by guaranteeing $\varepsilon_{\min}\left[n\right]\geq 1-\vartheta$ for a positive and arbitrarily small ϑ. Next, the condition for meeting the FWD’s and Bob’s required QoS is provided. Armed with the two conditions derived, an optimization problem maximizing the covert rate is established.

### 4.1. Performance Analysis

Using Equation (7), the probabilities of a false alarm and missed detection are calculated as

(11) $$P_{F}\left[n\right]=\left\{\begin{array}{l} 1,\lambda \left[n\right]<10^{\frac{\overset{⌣}{\delta }-\rho }{10}}\\ \frac{5}{\rho }\cdot \left[\frac{\overset{⌣}{\delta }+\rho }{10}-\frac{\ln\left(\lambda \left[n\right]\right)}{\ln10}\right],10^{\frac{\overset{⌣}{\delta }-\rho }{10}}\leq \lambda \left[n\right]<10^{\frac{\overset{⌣}{\delta }+\rho }{10}}\\ 0,\lambda \left[n\right]\geq 10^{\frac{\overset{⌣}{\delta }+\rho }{10}} \end{array}\right.$$

(12) $$P_{M}\left[n\right]=\left\{\begin{array}{l} 0,\lambda \left[n\right]<10^{\frac{\overset{⌣}{\delta }-\rho }{10}}+E_{W}\left[n\right]\\ \frac{5}{\rho }\left[\frac{\ln\left(\lambda \left[n\right]-E_{W}\left[n\right]\right)}{\ln10}-\frac{\overset{⌣}{\delta }-\rho }{10}\right],10^{\frac{\overset{⌣}{\delta }-\rho }{10}}+E_{W}\left[n\right]\leq \lambda \left[n\right]<10^{\frac{\overset{⌣}{\delta }+\rho }{10}}+E_{W}\left[n\right]\\ 1,\lambda \left[n\right]\geq 10^{\frac{\overset{⌣}{\delta }+\rho }{10}}+E_{W}\left[n\right] \end{array}\right.$$

where $E_{W}\left[n\right]=\beta_{0}P_{R}\left[n\right]/d_{\mathrm{RW}}^{2}$.

It can be observed from Equations (11) and (12) that $\varepsilon \left[n\right]$ may tend to 0 if $10^{\frac{\overset{⌣}{\delta }+\rho }{10}}<10^{\frac{\overset{⌣}{\delta }-\rho }{10}}+E_{W}\left[n\right]$ (i.e., $E_{W}\left[n\right]>10^{\frac{\overset{⌣}{\delta }+\rho }{10}}-10^{\frac{\overset{⌣}{\delta }-\rho }{10}}$ holds). Therefore, $E_{W}\left[n\right]\leq 10^{\frac{\overset{⌣}{\delta }+\rho }{10}}-10^{\frac{\overset{⌣}{\delta }-\rho }{10}}$ must be satisfied so that covert data delivery from R to Bob is feasible. If $E_{W}\left[n\right]\leq 10^{\frac{\overset{⌣}{\delta }+\rho }{10}}-10^{\frac{\overset{⌣}{\delta }-\rho }{10}}$, $\varepsilon \left[n\right]$ can be expressed as

(13) $$\varepsilon \left(n\right)=\left\{\begin{array}{l} 1,\lambda \left[n\right]<10^{\frac{\overset{⌣}{\delta }-\rho }{10}}\\ \frac{5}{\rho }\cdot \left[\frac{\overset{⌣}{\delta }+\rho }{10}-\frac{\ln\left(\lambda \left[n\right]\right)}{\ln10}\right],10^{\frac{\overset{⌣}{\delta }-\rho }{10}}\leq \lambda \left[n\right]<10^{\frac{\overset{⌣}{\delta }-\rho }{10}}+E_{W}\left[n\right]\\ 1-\frac{5}{\left(\ln10\right)\rho }\cdot \ln\left(\frac{\lambda \left[n\right]}{\lambda \left[n\right]-E_{W}\left[n\right]}\right),10^{\frac{\overset{⌣}{\delta }-\rho }{10}}+E_{W}\left[n\right]\leq \lambda \left[n\right]<10^{\frac{\overset{⌣}{\delta }+\rho }{10}}\\ \frac{5}{\rho }\left[\frac{\ln\left(\lambda \left[n\right]-E_{W}\left[n\right]\right)}{\ln10}-\frac{\overset{⌣}{\delta }-\rho }{10}\right],10^{\frac{\overset{⌣}{\delta }+\rho }{10}}\leq \lambda \left[n\right]<10^{\frac{\overset{⌣}{\delta }+\rho }{10}}+E_{W}\left[n\right]\\ 1,\lambda \left[n\right]\geq 10^{\frac{\overset{⌣}{\delta }+\rho }{10}}+E_{W}\left[n\right] \end{array}\right.$$

It can be proved that $\varepsilon \left[n\right]$ will achieve its minimum if the detection threshold $\lambda \left[n\right]$ is set to $10^{\frac{\overset{⌣}{\delta }-\rho }{10}}+E_{W}\left[n\right]$. Then, the minimum DEP $\varepsilon_{\min}\left[n\right]$ can be expressed as

(14) $$\varepsilon_{\min}\left[n\right]=\frac{5}{\rho }\cdot \left[\frac{\overset{⌣}{\delta }+\rho }{10}-\log\left(10^{\frac{\overset{⌣}{\delta }-\rho }{10}}+E_{W}\left[n\right]\right)\right].$$

To ensure covert data delivery from R to Bob, $\varepsilon_{\min}\left[n\right]\geq 1-\vartheta$ must be guaranteed for a positive and arbitrarily small ϑ. Then, substituting Equation (14) into $\varepsilon_{\min}\left[n\right]\geq 1-\vartheta$ leads to $E_{W}\left[n\right]\leq 10^{\frac{\rho \left(2\vartheta -1\right)+\overset{⌣}{\delta }}{10}}-10^{\frac{\overset{⌣}{\delta }-\rho }{10}}$. In addition, as mentioned above, covert data delivery from R to Bob requires that $E_{W}\left[n\right]\leq 10^{\frac{\overset{⌣}{\delta }+\rho }{10}}-10^{\frac{\overset{⌣}{\delta }-\rho }{10}}$ holds. Since $10^{\frac{\rho \left(2\vartheta -1\right)+\overset{⌣}{\delta }}{10}}-10^{\frac{\overset{⌣}{\delta }-\rho }{10}}<10^{\frac{\overset{⌣}{\delta }+\rho }{10}}-10^{\frac{\overset{⌣}{\delta }-\rho }{10}}$ holds and according to $E_{W}\left[n\right]=\beta_{0}P_{R}\left[n\right]/d_{\mathrm{RW}}^{2}$, the condition for ensuring covert data delivery from R to Bob can be expressed as $d_{\mathrm{RW}}\geq d_{{\mathrm{RW}}_{\min}}$, where $d_{{\mathrm{RW}}_{\min}}$ is the minimum distance that R must maintain from Willie and can be given by

(15) $$d_{{\mathrm{RW}}_{\min}}=\sqrt{\frac{\beta_{0}P_{R}\left[n\right]}{10^{\frac{\rho \left(1-2\vartheta \right)+\overset{⌣}{\delta }}{10}}-10^{\frac{\overset{⌣}{\delta }-\rho }{10}}}}$$

The preceding analysis explains that the FWD R must maintain a distance of at least $d_{{\mathrm{RW}}_{\min}}$ from Willie and $d_{{\mathrm{RW}}_{\min}}$ is proportional to its transmit power. In the following, $d_{\mathrm{RW}}\geq d_{{\mathrm{RW}}_{\min}}$ is referred to as covertness constraint.

If $d_{\mathrm{RW}}\geq d_{{\mathrm{RW}}_{\min}}$ is guaranteed, R can deliver covert data to Bob. In this situation, their required QoS should be met. Suppose that R delivers data at a covert rate of $R_{B}\left[n\right]$ in time-slot n. Then, Bob receives $y_{B}\left[n\right]=\sqrt{P_{R}\left[n\right]}h_{\mathrm{RB}}x_{R}\left[n\right]+n_{B}\left[n\right]$, where $n\in \left\{1,2,\cdots ,N\right\}$, $h_{\mathrm{RB}}$ is the channel gain between R and Bob and $n_{B}\left[n\right]$ is the noise with a power of $\delta_{B}$. Likewise, Bob has an uncertainty about the noise power $\delta_{B}$ and $h_{\mathrm{RB}}$ is assumed to follow the free space path loss model [11,18]. Then, $h_{\mathrm{RB}}$ can be written as $h_{\mathrm{RB}}=\sqrt{\beta_{0}/d_{\mathrm{RB}}^{2}}$, where $\beta_{0}$ denotes the channel power at unit distance and $d_{\mathrm{RB}}$ is the distance between R and Bob. At the end of time-slot n, the received signal-to-noise ratio (SNR) at Bob is $\gamma_{B}\left[n\right]=P_{R}\left[n\right]\beta_{0}/\left(\delta_{B}d_{\mathrm{RB}}^{2}\right)$ and then the achievable rate at Bob can be calculated as $\log_{2}\left(1+\gamma_{B}\left[n\right]\right)$ [11,18]. Due to the uncertainty about the noise power, $\delta_{B}$ is a random variable and its PDF can be given by Equation (7) as well.

In the article, outage probability is adopted as a measure of communication QoS. To meet the required QoS, Equation (16) must be satisfied:

(16) $$\mathrm{Pr}\left\{\log_{2}\left(1+\gamma_{B}\left[n\right]\right)<R_{B}\left[n\right]\right\}\leq \Theta_{B}.$$

In Equation (16), $\Theta_{B}$ is a design parameter defining the maximum outage probability that R and Bob can accept.

Using the PDF of $\delta_{B}$, the actual outage probability can be calculated as

(17) $$\begin{array}{l} \mathrm{Pr}\left\{\log_{2}\left(1+\gamma_{B}\left[n\right]\right)<R_{B}\left[n\right]\right\}=\mathrm{Pr}\left[\delta_{B}>\frac{P_{R}\left[n\right]\beta_{0}}{d_{\mathrm{RB}}^{2}\left(2^{R_{B}\left[n\right]}-1\right)}\right]\\ \;=\left\{\begin{array}{l} 1,\frac{P_{R}\left[n\right]\beta_{0}}{d_{\mathrm{RB}}^{2}\left(2^{R_{B}\left[n\right]}-1\right)}<10^{\frac{\overset{⌣}{\delta }-\rho }{10}}\\ \frac{5}{\rho }\left\{\frac{\overset{⌣}{\delta }+\rho }{10}-\log\left[\frac{P_{R}\left[n\right]\beta_{0}}{d_{\mathrm{RB}}^{2}\left(2^{R_{B}\left[n\right]}-1\right)}\right]\right\},10^{\frac{\overset{⌣}{\delta }-\rho }{10}}\leq \frac{P_{R}\left[n\right]\beta_{0}}{d_{\mathrm{RB}}^{2}\left(2^{R_{B}\left[n\right]}-1\right)}<10^{\frac{\overset{⌣}{\delta }+\rho }{10}}\\ 0,\frac{P_{R}\left[n\right]\beta_{0}}{d_{\mathrm{RB}}^{2}\left(2^{R_{B}\left[n\right]}-1\right)}\geq 10^{\frac{\overset{⌣}{\delta }+\rho }{10}} \end{array}\right.. \end{array}$$

It can be observed from Equation (17) that the actual outage probability can be classified as three exclusive cases and it is a non-decreasing function with respect to R’s transmit power $P_{R}\left[n\right]$. As revealed earlier, the minimum distance that R must maintain from Willie is proportional to $P_{R}\left[n\right]$, meaning that reducing $P_{R}\left[n\right]$ is beneficial for covert data delivery and for energy saving. On the other hand, Equation (16) must be satisfied so as to meet the required QoS, where $\Theta_{B}$ is a system design parameter and is typically set to 0.01 for high-quality personal communication systems [36]. Therefore, the actual outage probability should be kept below $\Theta_{B}$ when $10^{\frac{\overset{⌣}{\delta }-\rho }{10}}\leq P_{R}\left[n\right]\beta_{0}/d_{\mathrm{RB}}^{2}\left(2^{R_{B}\left[n\right]}-1\right)<10^{\frac{\overset{⌣}{\delta }+\rho }{10}}$ holds, which corresponds to the second case in Equation (17). Then, substituting Equation (17) into Equation (16) leads to $d_{\mathrm{RB}}\leq d_{{\mathrm{RB}}_{\max}}$, where $d_{{\mathrm{RB}}_{\max}}$ is the maximum distance that R can stay away from Bob and can be given by

(18) $$d_{{\mathrm{RB}}_{\max}}=\sqrt{\frac{P_{R}\left[n\right]\beta_{0}}{10^{\frac{\overset{⌣}{\delta }+\rho \left(1-2\Theta_{B}\right)}{10}}\left(2^{R_{B}\left[n\right]}-1\right)}}.$$

Therefore, to meet the required QoS, the FWD R must stay within $d_{{\mathrm{RB}}_{\max}}$ from Bob and $d_{{\mathrm{RB}}_{\max}}$ is proportional to its transmit power as well. In the following, $d_{\mathrm{RB}}\leq d_{{\mathrm{RB}}_{\max}}$ is referred to as outage constraint.

As mentioned earlier, the FWD R performs an unrestricted level flight within D with a radius of $r_{R}$ at a speed of v. Since the speed and the altitude remain constants, R either flies along a straight path or makes a banked level turn during its flight. In this situation, $v^{2}/gr\leq \tan\phi_{\max}$ must be satisfied [3], where g is the gravitational acceleration, r is the curvature radius of R’s flight path and $\phi_{\max}$ is R’s maximum bank angle limit. It should be noted that r is larger than 0 and will go to infinity when the FWD R flies along a straight path. Therefore, to ensure flight safety, the curvature radius of R’s flight path must satisfy $r\geq v^{2}/\left(g\tan\left|\phi_{\max}\right|\right)$.

### 4.2. Problem Establishment

Here, an optimization problem is established with the aim of maximizing the covert rate subject to the FWD’s mobility and transmit power limits as well as the derived covertness and outage constraints. This optimization problem can be written as

(19a) $$\left(x_{0}^{*},y_{0}^{*},h^{*},r_{R}^{*},P_{R}^{*}\left[n\right],v^{*}\right)\underset{x_{0},y_{0},h,r_{R},P_{R}\left[n\right],v}{=\arg\max}R_{B}\left[n\right]$$

(19b) $$s.t.\;{\left(x_{0}-r_{R}\cos\theta \right)}^{2}+{\left(y_{0}+r_{R}\sin\theta \right)}^{2}+h^{2}\geq d_{{\mathrm{RW}}_{\min}}^{2}$$

(19c) $${\left(d_{0}-x_{0}+r_{R}\cos\theta \right)}^{2}+{\left(y_{0}+r_{R}\sin\theta \right)}^{2}+h^{2}\leq d_{{\mathrm{RB}}_{\max}}^{2}$$

(19d) $$V_{\min}\leq v\leq V_{\max}$$

(19e) $$h_{\min}\leq h\leq h_{\max}$$

(19f) $$v^{2}/gr\leq \tan\phi_{\max},\;r>0$$

(19g) $$r_{R}\leq x_{0}\leq d_{0}-r_{R},\;0<r_{R}\leq d_{0}/2$$

(19h) $$q\left[n\right]\in D$$

(19i) $$P_{\min}\leq P_{R}\left[n\right]\leq P_{\max}$$

where $n\in \left\{1,2,\cdots ,N\right\}$, $\left(x_{0}^{*},y_{0}^{*},h^{*}\right)$ and $r_{R}^{*}$ are the optimal central position and radius of D, $P_{R}^{*}\left[n\right]$ and $v^{*}$ are the optimal transmit power and speed of the FWD R, θ is the central angle when R flies along the edge of D and can be any value in the interval of $\left[0^{\circ },360^{\circ }\right)$, and $q\left[n\right]$ denotes the position of R at time-slot n. Here, Equation (19b) ensures that the covertness constraint can be satisfied, where ${\left(x_{0}-r_{R}\cos\theta \right)}^{2}+{\left(y_{0}+r_{R}\sin\theta \right)}^{2}+h^{2}$ is the square of the distance from any point on the edge of D to Willie; Equation (19c) guarantees the satisfaction of the outage constraint, where ${\left(d_{0}-x_{0}+r_{R}\cos\theta \right)}^{2}+{\left(y_{0}+r_{R}\sin\theta \right)}^{2}+h^{2}$ denotes the square of the distance from any point on the edge of D to Bob. Equations (19d)~(19h) are R’s mobility constraints. Specifically, Equations (19d) and (19e) are R’s flight speed and altitude limits; Equation (19f) is R’s maximum bank angle limit; Equation (19g) gives the location and size restrictions of the circular area D; and Equation (19h) gives the restriction that R should be always within D. Equation (19i) gives R’s transmit power limit.

It should be noted that in problem (19), the FWD R is assumed to fly along the edge of the circular area D. Actually, R is capable of flying within D. Such an assumption aims to simplify the optimization problem due to the fact that as long as the covertness and outage constraints can be satisfied if R flies along the edge of D, they will be always met when R is within D.

## 5. Problem Solution and Algorithm Design

It can be observed from problem (19) that the global optimum can be obtained when the radius of D (i.e., $r_{R}$) achieves its minimum. From Equation (23), it can be observed that for fixed values of ρ, ϑ and $\Theta_{B}$, maximizing $d_{{\mathrm{RW}}_{\min}}/d_{{\mathrm{RB}}_{\max}}$ is equivalent to maximizing $R_{B}\left[n\right]$. Furthermore, according to the system model, when the ratio $d_{{\mathrm{RW}}_{\min}}/d_{{\mathrm{RB}}_{\max}}$ attains its maximum, the radius of the drone’s circular flight area D is minimized. Consequently, the global optimum is achieved at $r_{R}=r_{\min}$. To this end, the minimum radius of D is first derived and presented in the following propositions.

**Proposition** **1.** *The minimum radius of the circular area* D *, within which the FWD R is capable of performing an unrestricted level flight, can be given by*

(20) $$r_{R}^{*}=\frac{2V_{\min}^{2}}{g\tan\phi_{\max}}.$$

**Proof.**  When R performs a level flight, its maneuverability is reflected in its turning radius. The smaller the turning radius, the better the maneuverability will be. Therefore, to minimize its flight area, R should fly with its maximum maneuverability. As revealed in the preceding subsection, the curvature radius of R’s flight path must satisfy $r\geq v^{2}/\left(g\tan\left|\phi_{\max}\right|\right)$, and hence, the minimum turning radius is $r_{\min}=V_{\min}^{2}/g\tan\phi_{\max}$. It means that R should fly at its minimum speed with the maximum bank angle so as to achieve its maximum maneuverability. Suppose that R departs from $\left(x_{0},y_{0},h\right)$ with its maximum maneuverability. Then, the position of R at time t can be expressed as

(21) $$\begin{array}{ll} q\left(t\right) & =\left[x_{0}+V_{\min}\int_{0}^{t}\cos\left(\theta_{0}+\omega \tau \right)d\tau ,y_{0}+V_{\min}\int_{0}^{t}\sin\left(\theta_{0}+\omega \tau \right),h\right]\\ & =\left[x_{0}+\frac{V_{\min}}{\omega }\left[\sin\left(\theta_{0}+\omega t\right)-\sin\left(\theta_{0}\right)\right],y_{0}+\frac{V_{\min}}{\omega }\left[\cos\left(\theta_{0}\right)-\cos\left(\theta_{0}+\omega t\right)\right],h.\right] \end{array}$$

where $\theta_{0}$ is the directional angle of the speed and $\theta_{0}\in \left[0,2\pi \right)$ and $\omega =V_{\min}/r_{\min}$ is R’s angular velocity when utilizing its maximum maneuverability. At time t, the distance between R and its starting position can be calculated as $d\left(t\right)=V_{\min}\sqrt{2\left[1-\cos\left(\omega t\right)\right]}/\omega$. It is noticed that $d\left(t\right)$ will achieve its maximum value of $2V_{\min}/\omega$ when ωt is an odd multiple of π. It means that the longest distance that can be reached is $2r_{\min}$ when the FWD R departs from $\left(x_{0},y_{0},h\right)$ with its maximum maneuverability. On the other hand, suppose that R flies along a circle of radius $2r_{\min}$ at the speed $V_{\min}$. The FWD R is capable of starting from any point on the circle and then reaching the circle center with its maximum maneuverability. Therefore, the minimum radius of D, namely, the optimal radius of the circular region D for problem (19), is $2r_{\min}$. In addition, the optimal speed of R for problem (19) is $v^{*}=V_{\min}$. □

According to Proposition 1, problem (19) is infeasible if $r_{R}^{*}>d_{0}/2$, otherwise it can be transformed into

(22a) $$\left(x_{0}^{*},y_{0}^{*},h^{*},P_{R}^{*}\left[n\right]\right)\underset{x_{0},y_{0},h,P_{R}\left[n\right]}{=\arg\max}R_{B}\left[n\right]$$

(22b) $$s.t.\;{\left(x_{0}-r_{R}^{*}\cos\theta \right)}^{2}+{\left(y_{0}+r_{R}^{*}\sin\theta \right)}^{2}+h^{2}\geq d_{{\mathrm{RW}}_{\min}}^{2}$$

(22c) $${\left(d_{0}-x_{0}+r_{R}^{*}\cos\theta \right)}^{2}+{\left(y_{0}+r_{R}^{*}\sin\theta \right)}^{2}+h^{2}\leq d_{{\mathrm{RB}}_{\max}}^{2}$$

(22d) $$r_{R}^{*}\leq x_{0}\leq d_{0}-r_{R}^{*}$$

(22e) $$h_{\min}\leq h\leq h_{\max}$$

(22f) $$P_{\min}\leq P_{R}\left[n\right]\leq P_{\max}$$

where $r_{R}^{*}$ is given by Equation (20).

According to Equations (15) and (18), covert rate can be rewritten as

(23) $$R_{B}\left[n\right]=\log_{2}\left\{\frac{\left[10^{\frac{\rho \left(1-2\vartheta \right)+\overset{⌣}{\delta }}{10}}-10^{\frac{\overset{⌣}{\delta }-\rho }{10}}\right]}{10^{\frac{\overset{⌣}{\delta }+\rho \left(1-2\Theta_{B}\right)}{10}}}\cdot \frac{d_{{\mathrm{RW}}_{\min}}^{2}}{d_{{\mathrm{RB}}_{\max}}^{2}}+1\right\}.$$

It can be observed from Equation (23) that maximizing $R_{B}\left[n\right]$ is equivalent to maximizing the ratio $d_{{\mathrm{RW}}_{\min}}^{2}/d_{{\mathrm{RB}}_{\max}}^{2}$. Thus, the maximum covert rate will be achieved when $d_{{\mathrm{RW}}_{\min}}^{2}/d_{{\mathrm{RB}}_{\max}}^{2}$ attains its maximum. It means that the global maximum will be achieved when the inequality constraints given by Equations (22b) and (22c) hold with equality.

As revealed in the preceding section, the FWD R should stay as far away from Willie as possible so as to satisfy the covertness constraint. In addition, the minimum distance $d_{{\mathrm{RW}}_{\min}}$ that R should stay away from Willie is proportional to its transmit power $P_{R}\left[n\right]$. Thus, replacing $P_{R}\left[n\right]$ with $P_{\min}$ or $P_{\max}$ in Equation (15) yields the lower-bound or upper-bound of $d_{{\mathrm{RW}}_{\min}}$. In the following, the lower-bound and upper-bound of $d_{{\mathrm{RW}}_{\min}}$ are denoted as $d_{{\mathrm{RW}}_{\min}}^{\mathrm{LB}}$ and $d_{{\mathrm{RW}}_{\min}}^{\mathrm{UB}}$, respectively.

Likewise, the FWD R should approach Bob as closely as possible so as to meet the required QoS and the maximum distance that R can stay away from Bob is $d_{{\mathrm{RB}}_{\max}}$. Since R’s minimum flight altitude is $h_{\min}$ and the optimal radius of D is $r_{R}^{*}$, the theoretical minimum value of $d_{{\mathrm{RB}}_{\max}}$ is $\sqrt{h_{\min}^{2}+4{\left(r_{R}^{*}\right)}^{2}}$, which is denoted as $d_{{\mathrm{RB}}_{\max}}^{\mathrm{LB}}$ in this context. In this situation, the coordinate of the central position of D is $\left(d_{0}-r_{R}^{*},0,h_{\min}\right)$. For ease of presentation, the above case is referred to as the nearest location of D.

According to the above analyses, the solution of problem (22) can be classified as the following two cases.

Case 1: $\sqrt{{\left(d_{{\mathrm{RW}}_{\min}}^{\mathrm{UB}}\right)}^{2}-h_{\min}^{2}}+2r_{R}^{*}\leq d_{0}$ holds. In this situation, D and $x^{2}+y^{2}+z^{2}={\left(d_{{\mathrm{RW}}_{\min}}^{\mathrm{UB}}\right)}^{2}$ will not intersect or be tangent when D situates in the nearest location. To maximize the covert rate, the FWD R should delivery data with its maximum transmit power and fly within D with the central position of $\left(d_{0}-r_{R}^{*},0,h_{\min}\right)$, meaning that $P_{R}^{*}\left[n\right]=P_{\max}$, $x_{0}^{*}=d_{0}-r_{R}^{*}$, $y_{0}^{*}=0$ and $h^{*}=h_{\min}$. Then, the maximum cover rate can be given by

(24) $$R_{B}\left[n\right]=\log_{2}\left\{1+\frac{\beta_{0}P_{\max}}{10^{\frac{\overset{⌣}{\delta }+\rho \left(1-2\Theta_{B}\right)}{10}}{\left(d_{{\mathrm{RB}}_{\max}}^{\mathrm{LB}}\right)}^{2}}\right\},$$

where $d_{{\mathrm{RB}}_{\max}}^{\mathrm{LB}}=\sqrt{h_{\min}^{2}+4{\left(r_{R}^{*}\right)}^{2}}$ is the theoretical minimum value of $d_{{\mathrm{RB}}_{\max}}$.

Case 2: $\sqrt{{\left(d_{{\mathrm{RW}}_{\min}}^{\mathrm{UB}}\right)}^{2}-h_{\min}^{2}}+2r_{R}^{*}>d_{0}$ holds. In this situation, D and $x^{2}+y^{2}+z^{2}={\left(d_{{\mathrm{RW}}_{\min}}^{\mathrm{UB}}\right)}^{2}$ will intersect when D is situated in the nearest location and the coordinate along the z-axis of the intersection point is larger than $h_{\min}$. As revealed earlier, maximizing $R_{B}\left[n\right]$ is equivalent to maximizing $d_{{\mathrm{RW}}_{\min}}^{2}/d_{{\mathrm{RB}}_{\max}}^{2}$, and the maximum $R_{B}\left[n\right]$ will be achieved when $d_{{\mathrm{RW}}_{\min}}^{2}/d_{{\mathrm{RB}}_{\max}}^{2}$ attains its maximum. It can be deduced that if D and $x^{2}+y^{2}+z^{2}=d_{{\mathrm{RW}}_{\min}}^{2}$ are tangent at $x=d_{0}-2r_{R}^{*}$, $d_{{\mathrm{RW}}_{\min}}^{2}/d_{{\mathrm{RB}}_{\max}}^{2}$ achieves its maximum. In this situation, the projection of the central position of D is on the line between Willie and Bob. Then, $d_{{\mathrm{RW}}_{\min}}^{2}/d_{{\mathrm{RB}}_{\max}}^{2}$ can be written as

(25) $$\frac{d_{{\mathrm{RW}}_{\min}}^{2}}{d_{{\mathrm{RB}}_{\max}}^{2}}=\frac{h^{2}+{\left(d_{0}-2r_{R}^{*}\right)}^{2}}{h^{2}+4{\left(r_{R}^{*}\right)}^{2}}.$$

Obviously, Equation (25) is a decreasing function of h if $d_{0}>4r_{R}^{*}$, while it is an increasing function of h if $2r_{R}^{*}<d_{0}\leq 4r_{R}^{*}$. Therefore, R should fly as low as possible when $d_{0}>4r_{R}^{*}$ holds, while R should fly as high as possible when $2r_{R}^{*}<d_{0}\leq 4r_{R}^{*}$ holds such that covert data delivered can be carried out and the required QoS can be met. To solve the optimization problem, the following six sub-cases are examined.

Sub-case 2.1: $d_{0}>4r_{R}^{*}$ and $\sqrt{{\left(d_{{\mathrm{RW}}_{\min}}^{\mathrm{LB}}\right)}^{2}-{\left(d_{0}-2r_{R}^{*}\right)}^{2}}<h_{\min}$ hold. In this situation, the FWD R should fly as low as possible, and D can be tangent to $x^{2}+y^{2}+z^{2}=d_{{\mathrm{RW}}_{\min}}^{2}$ when D is situated in the nearest location. This means that $h^{*}=h_{\min}$, $x_{0}^{*}=d_{0}-r_{R}^{*}$, $y_{0}^{*}=0$, $d_{{\mathrm{RB}}_{\max}}=\sqrt{4{\left(r_{R}^{*}\right)}^{2}+h_{\min}^{2}}$ and $d_{{\mathrm{RW}}_{\min}}=\sqrt{h_{\min}^{2}+{\left(d_{0}-2r_{R}^{*}\right)}^{2}}$. Using $d_{{\mathrm{RW}}_{\min}}=\sqrt{h_{\min}^{2}+{\left(d_{0}-2r_{R}^{*}\right)}^{2}}$ and Equation (15), the optimal transmit power of R can be obtained, giving

(26) $$P_{R}^{*}\left[n\right]=\frac{\left[h_{\min}^{2}+{\left(d_{0}-2r_{R}^{*}\right)}^{2}\right]\left[10^{\frac{\rho \left(1-2\vartheta \right)+\overset{⌣}{\delta }}{10}}-10^{\frac{\overset{⌣}{\delta }-\rho }{10}}\right]}{\beta_{0}}.$$

Using $d_{{\mathrm{RB}}_{\max}}=\sqrt{4{\left(r_{R}^{*}\right)}^{2}+h_{\min}^{2}}$ and Equations (18) and (26), the maximum covert rate can be calculated as

(27) $$R_{B}\left[n\right]=\log_{2}\left\{1+\frac{\left[h_{\min}^{2}+{\left(d_{0}-2r_{R}^{*}\right)}^{2}\right]\left[10^{\frac{\rho \left(1-2\vartheta \right)+\overset{⌣}{\delta }}{10}}-10^{\frac{\overset{⌣}{\delta }-\rho }{10}}\right]}{10^{\frac{\overset{⌣}{\delta }+\rho \left(1-2\Theta_{B}\right)}{10}}\left[4{\left(r_{R}^{*}\right)}^{2}+h_{\min}^{2}\right]}\right\}.$$

Sub-case 2.2: $d_{0}>4r_{R}^{*}$ and $h_{\min}\leq \sqrt{{\left(d_{{\mathrm{RW}}_{\min}}^{\mathrm{LB}}\right)}^{2}-{\left(d_{0}-2r_{R}^{*}\right)}^{2}}\leq h_{\max}$ hold. Here, $d_{0}>4r_{R}^{*}$ implies that the drone should fly as low as possible, while $h_{\min}\leq \sqrt{{\left(d_{{\mathrm{RW}}_{\min}}^{\mathrm{LB}}\right)}^{2}-{\left(d_{0}-2r_{R}^{*}\right)}^{2}}\leq h_{\max}$ means that the drone can fly with an altitude between $h_{\min}$ and $h_{\max}$ when it is exactly $d_{{\mathrm{RW}}_{\min}}^{\mathrm{LB}}$ away from Willie. Thus, the drone can only use its minimum power $P_{\min}$ to transmit data. In this situation, R should fly as low as possible, and D can be tangent to $x^{2}+y^{2}+z^{2}=d_{{\mathrm{RW}}_{\min}}^{2}$ when $h_{\min}\leq h\leq h_{\max}$. Therefore, R should operate in its minimum transmit power such that it is capable of flying at the lowest altitude. It means that $P_{R}^{*}\left[n\right]=P_{\min}$, $h^{*}=\sqrt{{\left(d_{{\mathrm{RW}}_{\min}}^{\mathrm{LB}}\right)}^{2}-{\left(d_{0}-2r_{R}^{*}\right)}^{2}}$, $x_{0}^{*}=d_{0}-r_{R}^{*}$ and $y_{0}^{*}=0$. Then, $d_{{\mathrm{RB}}_{\max}}=\sqrt{4{\left(r_{R}^{*}\right)}^{2}+{\left(h^{*}\right)}^{2}}$ can be obtained. Using $d_{{\mathrm{RB}}_{\max}}=\sqrt{4{\left(r_{R}^{*}\right)}^{2}+{\left(h^{*}\right)}^{2}}$ and Equation (18), the maximum covert rate can be calculated as

(28) $$R_{B}\left[n\right]=\log_{2}\left\{1+\frac{\beta_{0}P_{\min}}{10^{\frac{\overset{⌣}{\delta }+\rho \left(1-2\Theta_{B}\right)}{10}}\left[4{\left(r_{R}^{*}\right)}^{2}+h^{2}\right]}\right\}.$$

Sub-case 2.3: $d_{0}>4r_{R}^{*}$ and $\sqrt{{\left(d_{{\mathrm{RW}}_{\min}}^{\mathrm{LB}}\right)}^{2}-{\left(d_{0}-2r_{R}^{*}\right)}^{2}}\geq h_{\max}$ hold. Here, $d_{0}>4r_{R}^{*}$ implies that the drone should fly as low as possible, while $\sqrt{{\left(d_{{\mathrm{RW}}_{\min}}^{\mathrm{LB}}\right)}^{2}-{\left(d_{0}-2r_{R}^{*}\right)}^{2}}\geq h_{\max}$ means that the drone’s flight altitude exceeds the maximum limit, even when it is exactly $d_{{\mathrm{RW}}_{\min}}^{\mathrm{LB}}$ away from Willie. In this situation, the drone remains exactly $d_{{\mathrm{RW}}_{\min}}^{\mathrm{LB}}$ away from Willie and can therefore only use its minimum power $P_{\min}$ to transmit data, i.e., $P_{R}^{*}\left[n\right]=P_{\min}$. In addition, D can only be tangent to $x^{2}+y^{2}+z^{2}=d_{{\mathrm{RW}}_{\min}}^{2}$ when $h>h_{\max}$, and hence, the projection of the central position of D cannot be on the line between Willie and Bob. To meet the flight altitude limit, D needs to move along the y-axis until R’s flight altitude is equal to $h_{\max}$, i.e., $h^{*}=h_{\max}$. Then, $x_{0}^{*}=d_{0}-r_{R}^{*}$ and $y_{0}^{*}=\pm \sqrt{{\left[\sqrt{{\left(d_{{\mathrm{RW}}_{\min}}^{\mathrm{LB}}\right)}^{2}-h_{\max}^{2}}+r_{R}^{*}\right]}^{2}-{\left(d_{0}-r_{R}^{*}\right)}^{2}}$ can be obtained followed by the calculation of $d_{{\mathrm{RB}}_{\max}}=\sqrt{{\left[\sqrt{{\left(x_{0}^{*}-d_{0}\right)}^{2}+{\left(y_{0}^{*}\right)}^{2}}+r_{R}^{*}\right]}^{2}+h_{\max}^{2}}$. Using $d_{{\mathrm{RB}}_{\max}}$ and Equation (18), the maximum covert rate can be calculated as

(29) $$R_{B}\left[n\right]=\log_{2}\left\{1+\frac{\beta_{0}P_{\min}}{10^{\frac{\overset{⌣}{\delta }+\rho \left(1-2\Theta_{B}\right)}{10}}\left({\left[\sqrt{{\left(x_{0}-d_{0}\right)}^{2}+y_{0}^{2}}+r_{R}^{*}\right]}^{2}+h_{\max}^{2}\right)}\right\}.$$

Sub-case 2.4: $2r_{R}^{*}<d_{0}\leq 4r_{R}^{*}$ and $\sqrt{{\left(d_{{\mathrm{RW}}_{\min}}^{\mathrm{UB}}\right)}^{2}-{\left(d_{0}-2r_{R}^{*}\right)}^{2}}\leq h_{\max}$ hold. As revealed earlier, R needs to fly as high as possible in this sub-case. Then, R should operate in its maximum transmit power, i.e., $P_{R}^{*}\left[n\right]=P_{\max}$ and the optimal flight altitude is $h^{*}=\sqrt{{\left(d_{{\mathrm{RW}}_{\min}}^{\mathrm{UB}}\right)}^{2}-{\left(d_{0}-2r_{R}^{*}\right)}^{2}}$, which leads to $x_{0}^{*}=d_{0}-r_{R}^{*}$ and $y_{0}^{*}=0$. Then, $d_{{\mathrm{RB}}_{\max}}$ can be calculated as $d_{{\mathrm{RB}}_{\max}}=\sqrt{{\left(h^{*}\right)}^{2}+4{\left(r_{R}^{*}\right)}^{2}}$. According to $d_{{\mathrm{RB}}_{\max}}$ and Equation (18), the maximum covert rate can be calculated as

(30) $$R_{B}\left[n\right]=\log_{2}\left\{1+\frac{\beta_{0}P_{\max}}{10^{\frac{\overset{⌣}{\delta }+\rho \left(1-2\Theta_{B}\right)}{10}}\left[{\left(d_{{\mathrm{RW}}_{\min}}^{\mathrm{UB}}\right)}^{2}-{\left(d_{0}-2r_{R}^{*}\right)}^{2}+4{\left(r_{R}^{*}\right)}^{2}\right]}\right\}.$$

Sub-case 2.5: $2r_{R}^{*}<d_{0}\leq 4r_{R}^{*}$, $\sqrt{{\left(d_{{\mathrm{RW}}_{\min}}^{\mathrm{UB}}\right)}^{2}-{\left(d_{0}-2r_{R}^{*}\right)}^{2}}>h_{\max}$ and $\sqrt{{\left(d_{{\mathrm{RW}}_{\min}}^{\mathrm{LB}}\right)}^{2}-{\left(d_{0}-2r_{R}^{*}\right)}^{2}}\leq h_{\max}$ hold. In this situation, the optimal flight altitude of R is $h^{*}=h_{\max}$ and then $x_{0}^{*}=d_{0}-r_{R}^{*}$ and $y_{0}^{*}=0$ can be obtained straightforwardly. Then, $d_{{\mathrm{RB}}_{\max}}$ and $d_{{\mathrm{RW}}_{\min}}$ can be calculated as $d_{{\mathrm{RB}}_{\max}}=\sqrt{h_{\max}^{2}+4{\left(r_{R}^{*}\right)}^{2}}$ and $d_{{\mathrm{RW}}_{\min}}=\sqrt{h_{\max}^{2}+{\left(d_{0}-2r_{R}^{*}\right)}^{2}}$. According to $d_{{\mathrm{RW}}_{\min}}$ and Equation (15), the optimal transmit power of the FWD R is

(31) $$P_{R}^{*}\left[n\right]=\frac{\left[h_{\max}^{2}+{\left(d_{0}-2r_{R}^{*}\right)}^{2}\right]\left[10^{\frac{\rho \left(1-2\vartheta \right)+\overset{⌣}{\delta }}{10}}-10^{\frac{\overset{⌣}{\delta }-\rho }{10}}\right]}{\beta_{0}}.$$

According to $d_{{\mathrm{RB}}_{\max}}$ and Equations (18) and (31), the maximum covert rate can be calculated as

(32) $$R_{B}\left[n\right]=\log_{2}\left\{1+\frac{\left[h_{\max}^{2}+{\left(d_{0}-2r_{R}^{*}\right)}^{2}\right]\left[10^{\frac{\rho \left(1-2\vartheta \right)+\overset{⌣}{\delta }}{10}}-10^{\frac{\overset{⌣}{\delta }-\rho }{10}}\right]}{10^{\frac{\overset{⌣}{\delta }+\rho \left(1-2\Theta_{B}\right)}{10}}\left[h_{\max}^{2}+4{\left(r_{R}^{*}\right)}^{2}\right]}\right\}.$$

Sub-case 2.6: $2r_{R}^{*}<d_{0}\leq 4r_{R}^{*}$ and $\sqrt{{\left(d_{{\mathrm{RW}}_{\min}}^{\mathrm{LB}}\right)}^{2}-{\left(d_{0}-2r_{R}^{*}\right)}^{2}}>h_{\max}$ hold. Here, $2r_{R}^{*}<d_{0}\leq 4r_{R}^{*}$ implies that the drone should fly as high as possible, while $\sqrt{{\left(d_{{\mathrm{RW}}_{\min}}^{\mathrm{LB}}\right)}^{2}-{\left(d_{0}-2r_{R}^{*}\right)}^{2}}>h_{\max}$ means that the drone’s flight altitude exceeds the maximum limit, even when it is exactly $d_{{\mathrm{RW}}_{\min}}^{\mathrm{LB}}$ away from Willie. In this situation, D can only be tangent to $x^{2}+y^{2}+z^{2}=d_{{\mathrm{RW}}_{\min}}^{2}$ when $h>h_{\max}$ and hence, R can only operate in the minimum transmit power, i.e., $P_{R}^{*}\left[n\right]=P_{\min}$. To meet the flight altitude limit, D needs to move along the y-axis until R’s flight altitude is equal to $h_{\max}$, i.e., $h^{*}=h_{\max}$. Then, $x_{0}^{*}=d_{0}-r_{R}^{*}$ and $y_{0}^{*}=\pm \sqrt{{\left[\sqrt{{\left(d_{{\mathrm{RW}}_{\min}}^{\mathrm{LB}}\right)}^{2}-h_{\max}^{2}}+r_{R}^{*}\right]}^{2}-{\left(d_{0}-r_{R}^{*}\right)}^{2}}$ can be obtained, followed by the calculation of $d_{{\mathrm{RB}}_{\max}}=\sqrt{{\left[\sqrt{{\left(x_{0}^{*}-d_{0}\right)}^{2}+{\left(y_{0}^{*}\right)}^{2}}+r_{R}^{*}\right]}^{2}+h_{\max}^{2}}$. According to $d_{{\mathrm{RB}}_{\max}}$ and Equation (18), the maximum covert rate can be calculated and given by Equation (29).

Summarizing the above analyses, an optimization algorithm is presented as below.
**Algorithm 1**: Joint optimization algorithm for carrying out covert data delivery with the maximum covert rate, while meeting the required QoS.Step 1: Initialize the system parameters, including the gravitational acceleration g, Willie and Bob’s 3D coordinates $q_{w}$ and $q_{B}$, the FWD’s minimum and maximum flight altitudes $h_{\min}$ and $h_{\max}$, the FWD’s minimum and maximum flight speeds $V_{\min}$ and $V_{\max}$, the FWD’s minimum and maximum transmit power $P_{\min}$ and $P_{\max}$, the FWD’s maximum bank angle $\phi_{\max}$, the channel power at unit distance $\beta_{0}$, the positive and arbitrarily small parameter ϑ, the outage probability limit $\Theta_{B}$, the mean value of the noise power in the dB domain $\overset{⌣}{\delta }$ and the noise uncertainty parameter in the dB domain ρ.
Step 2: Calculate the distance between Willie and Bob $d_{0}=\left\|q_{w}-q_{B}\right\|$, $d_{{\mathrm{RW}}_{\min}}^{\mathrm{LB}}$ and $d_{{\mathrm{RW}}_{\min}}^{\mathrm{UB}}$. According to Equation (20), calculate the optimal radius $r_{R}^{*}$ and $d_{{\mathrm{RB}}_{\max}}^{\mathrm{LB}}=\sqrt{h_{\min}^{2}+4{\left(r_{R}^{*}\right)}^{2}}$. Set the optimal flight speed as $v^{*}=V_{\min}$.
Step 3: If $\sqrt{{\left(d_{{\mathrm{RW}}_{\min}}^{\mathrm{UB}}\right)}^{2}-h_{\min}^{2}}+2r_{R}^{*}\leq d_{0}$, the FWD’s optimal transmit power is $P_{R}^{*}\left[n\right]=P_{\max}$, $x_{0}^{*}=d_{0}-r_{R}^{*}$, $y_{0}^{*}=0$ and $h^{*}=h_{\min}$. According to Equation (24), calculate the maximum cover rate. Next, turn to Step 11.
Step 4: If $d_{0}>4r_{R}^{*}$ and $\sqrt{{\left(d_{{\mathrm{RW}}_{\min}}^{\mathrm{LB}}\right)}^{2}-{\left(d_{0}-2r_{R}^{*}\right)}^{2}}<h_{\min}$, $x_{0}^{*}=d_{0}-r_{R}^{*}$, $y_{0}^{*}=0$ and $h^{*}=h_{\min}$. According to Equations (26) and (27), calculate the optimal transmit power $P_{R}^{*}\left[n\right]$ and the maximum cover rate, respectively. Next, turn to Step 11.
Step 5: If $d_{0}>4r_{R}^{*}$ and $h_{\min}\leq \sqrt{{\left(d_{{\mathrm{RW}}_{\min}}^{\mathrm{LB}}\right)}^{2}-{\left(d_{0}-2r_{R}^{*}\right)}^{2}}\leq h_{\max}$, the FWD’s optimal transmit power is $P_{R}^{*}\left[n\right]=P_{\min}$, and $x_{0}^{*}=d_{0}-r_{R}^{*}$, $y_{0}^{*}=0$, $h^{*}=\sqrt{{\left(d_{{\mathrm{RW}}_{\min}}^{\mathrm{LB}}\right)}^{2}-{\left(d_{0}-2r_{R}^{*}\right)}^{2}}$. According to Equation (28), calculate the maximum covert rate. Next, turn to Step 11.
Step 6: If $d_{0}>4r_{R}^{*}$ and $\sqrt{{\left(d_{{\mathrm{RW}}_{\min}}^{\mathrm{LB}}\right)}^{2}-{\left(d_{0}-2r_{R}^{*}\right)}^{2}}\geq h_{\max}$, the FWD’s optimal transmit power is $P_{R}^{*}\left[n\right]=P_{\min}$ and $x_{0}^{*}=d_{0}-r_{R}^{*}$, $y_{0}^{*}=\pm \sqrt{{\left[\sqrt{{\left(d_{{\mathrm{RW}}_{\min}}^{\mathrm{LB}}\right)}^{2}-h_{\max}^{2}}+r_{R}^{*}\right]}^{2}-{\left(d_{0}-r_{R}^{*}\right)}^{2}}$, $h^{*}=h_{\max}$. According to Equation (29), calculate the maximum covert rate. Next, turn to Step 11.
Step 7: If $2r_{R}^{*}<d_{0}\leq 4r_{R}^{*}$ and $\sqrt{{\left(d_{{\mathrm{RW}}_{\min}}^{\mathrm{UB}}\right)}^{2}-{\left(d_{0}-2r_{R}^{*}\right)}^{2}}\leq h_{\max}$, the FWD’s optimal transmit power is $P_{R}^{*}\left[n\right]=P_{\max}$ and $x_{0}^{*}=d_{0}-r_{R}^{*}$, $y_{0}^{*}=0$, $h^{*}=\sqrt{{\left(d_{{\mathrm{RW}}_{\min}}^{\mathrm{UB}}\right)}^{2}-{\left(d_{0}-2r_{R}^{*}\right)}^{2}}$. According to Equation (30), calculate the maximum covert rate. Next, turn to Step 11.
Step 8: If $2r_{R}^{*}<d_{0}\leq 4r_{R}^{*}$, $\sqrt{{\left(d_{{\mathrm{RW}}_{\min}}^{\mathrm{UB}}\right)}^{2}-{\left(d_{0}-2r_{R}^{*}\right)}^{2}}>h_{\max}$ and $\sqrt{{\left(d_{{\mathrm{RW}}_{\min}}^{\mathrm{LB}}\right)}^{2}-{\left(d_{0}-2r_{R}^{*}\right)}^{2}}\leq h_{\max}$, $x_{0}^{*}=d_{0}-r_{R}^{*}$, $y_{0}^{*}=0$ and $h^{*}=h_{\max}$. According to Equations (31) and (32), calculate the optimal transmit power $P_{R}^{*}\left[n\right]$ and the maximum covert rate, respectively. Next, turn to Step 11.
Step 9: If $2r_{R}^{*}<d_{0}\leq 4r_{R}^{*}$ and $\sqrt{{\left(d_{{\mathrm{RW}}_{\min}}^{\mathrm{LB}}\right)}^{2}-{\left(d_{0}-2r_{R}^{*}\right)}^{2}}>h_{\max}$, the FWD’s optimal transmit power is $P_{R}^{*}\left[n\right]=P_{\min}$ and $x_{0}^{*}=d_{0}-r_{R}^{*}$, $y_{0}^{*}=\pm \sqrt{{\left[\sqrt{{\left(d_{{\mathrm{RW}}_{\min}}^{\mathrm{LB}}\right)}^{2}-h_{\max}^{2}}+r_{R}^{*}\right]}^{2}-{\left(d_{0}-r_{R}^{*}\right)}^{2}}$, $h^{*}=h_{\max}$. According to Equation (29), calculate the maximum covert rate. Next, turn to Step 11.
Step 10: If $d_{0}\leq 2r_{R}^{*}$, the FWD and Bob stay silent and do not deliver any data. Next, turn to Step 12.
Step 11: The FWD performs an unrestricted level flight within the circular area D with the central position $\left(x_{0}^{*},y_{0}^{*},h^{*}\right)$ and the radius $r_{R}^{*}$ at the speed $v^{*}$ and meanwhile the FWD delivers data to Bob at the calculated covert rate at any time. Step 12: The algorithm ends.

## 6. Simulation Results

In this section, computer simulations are conducted to verify the correctness and effectiveness of the proposed optimization algorithm. Table 1 gives the values of the parameters used in the simulations [2,10,36].

In the simulations, three different noise uncertainty cases are involved, where the noise uncertainty parameter in the dB domain is set to ρ=0.5 dB, ρ=1 dB and ρ=1.5 dB, respectively. It should be noted that the larger the value of ρ, the greater the uncertainty about noise power will be. In addition, the distance between Willie and Bob ranges between 15 and 500 m. To verify the correctness and efficiency of the proposed **Algorithm 1**, the sequential quadratic programming available in the MATLABR2024b Optimization Toolbox is used to solve the initial optimization problem and the achieved results are denoted as “simulation” in the plotted Figures.

It should be noted that according to Equation (20), the minimum radius of D is $r_{R}^{*}=5.102\;m$ as $V_{\min}=5\;m/s$ and $\phi_{\max}=45^{\circ }$ are assumed. As revealed in preceding section, the FWD should fly as low as possible if $d_{0}>20.408$, while it should fly as high as possible if $10.204<d_{0}\leq 20.408$.

**Figure 2:**
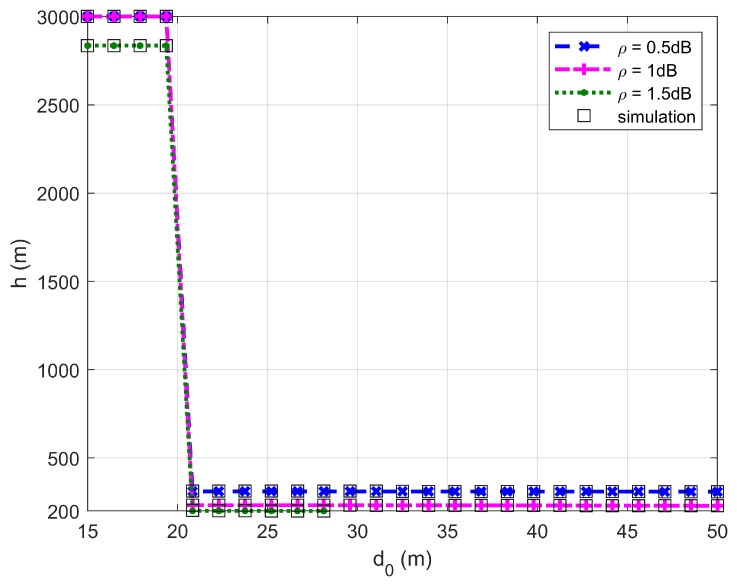
Flight altitude versus $d_{0}$, where $d_{0}$ ranges from 15 to 50 m.

**Figure 3:**
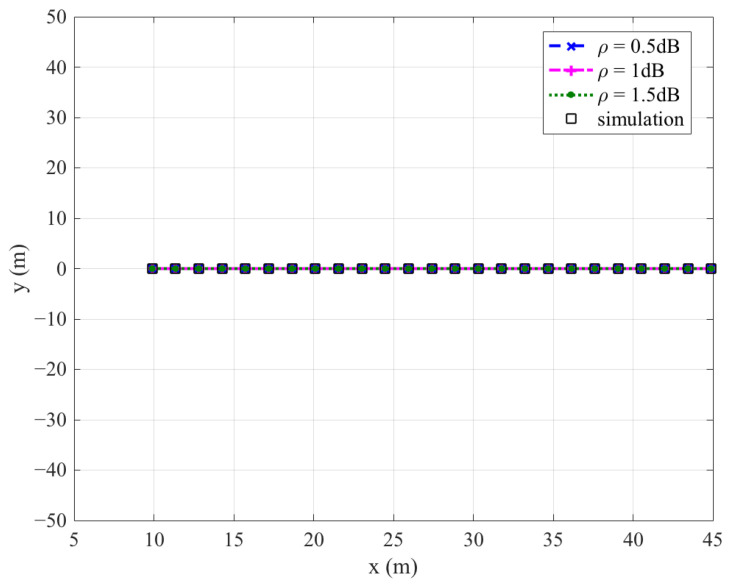
Horizontal coordinate of the central position of D versus $d_{0}$, where $d_{0}$ ranges from 15 to 50 m.

**Figure 4:**
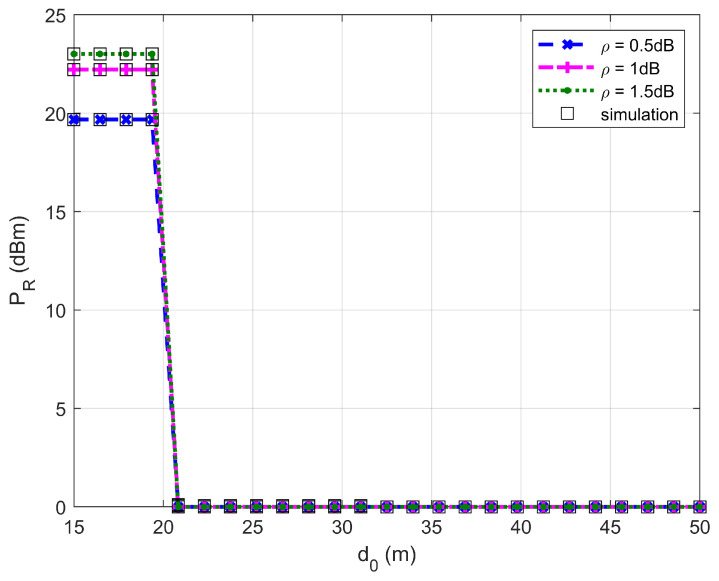
Transmit power versus $d_{0}$, where $d_{0}$ ranges from 15 to 50 m.

**Figure 5:**
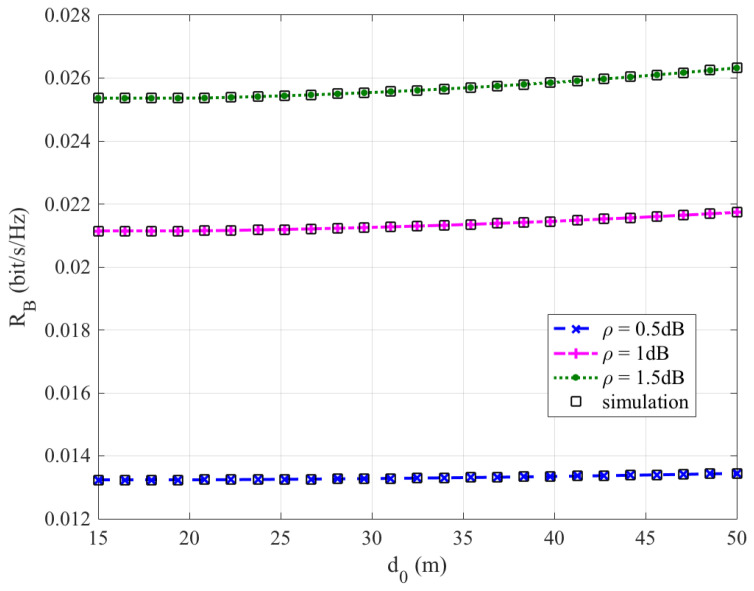
Covert rate versus $d_{0}$, where $d_{0}$ ranges from 15 to 50 m.

**Figure 6:**
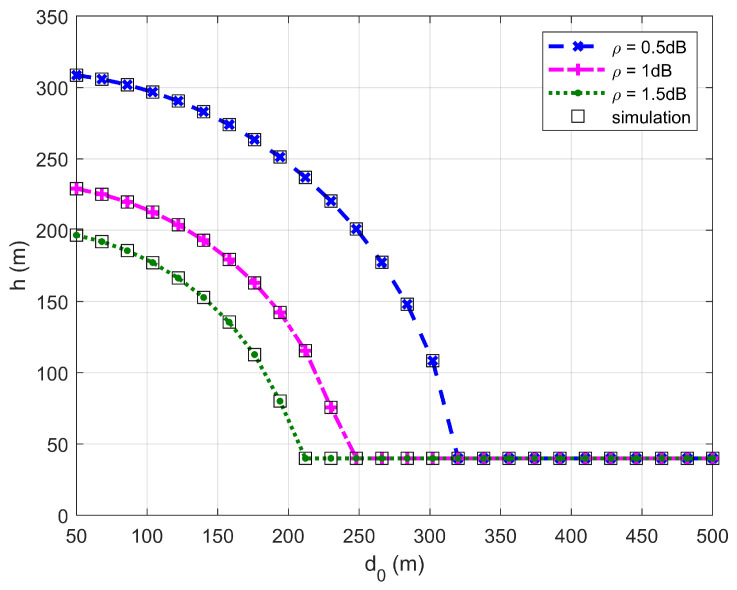
Flight altitude versus $d_{0}$, where $d_{0}$ ranges from 50 to 500 m.

**Figure 7:**
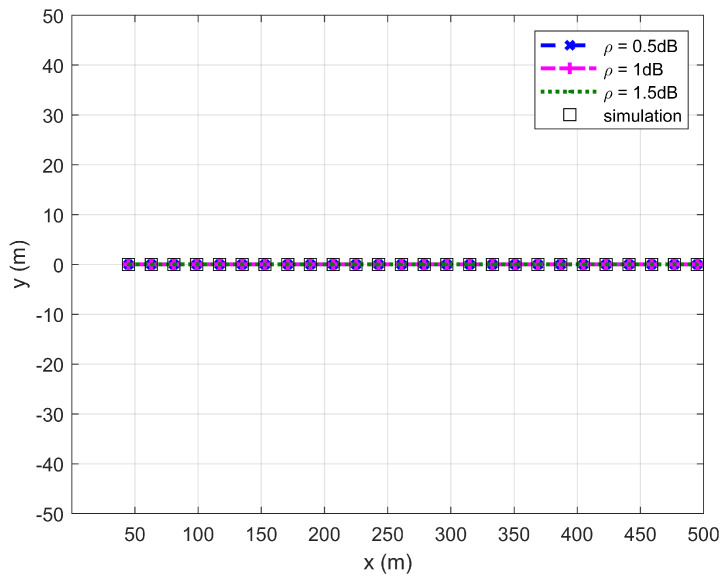
Horizontal coordinate of the central position of D versus $d_{0}$, where $d_{0}$ ranges from 50 to 500 m.

**Figure 8:**
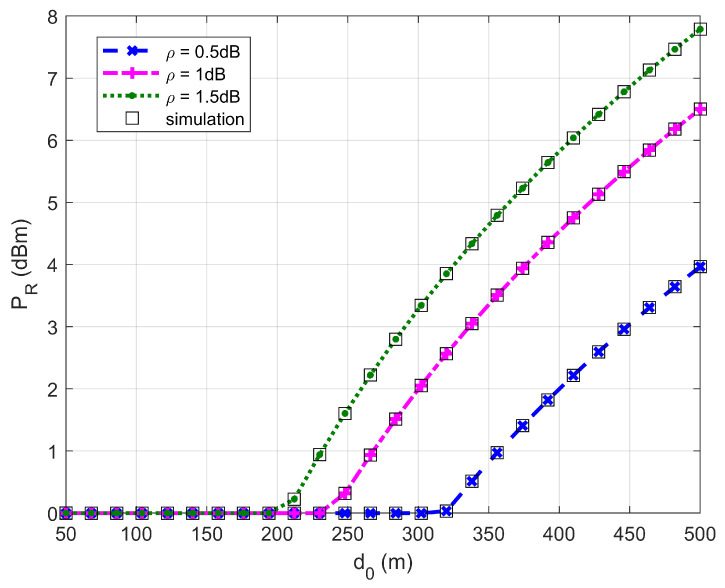
Transmit power versus $d_{0}$, where $d_{0}$ ranges from 50 to 500 m.

**Figure 9:**
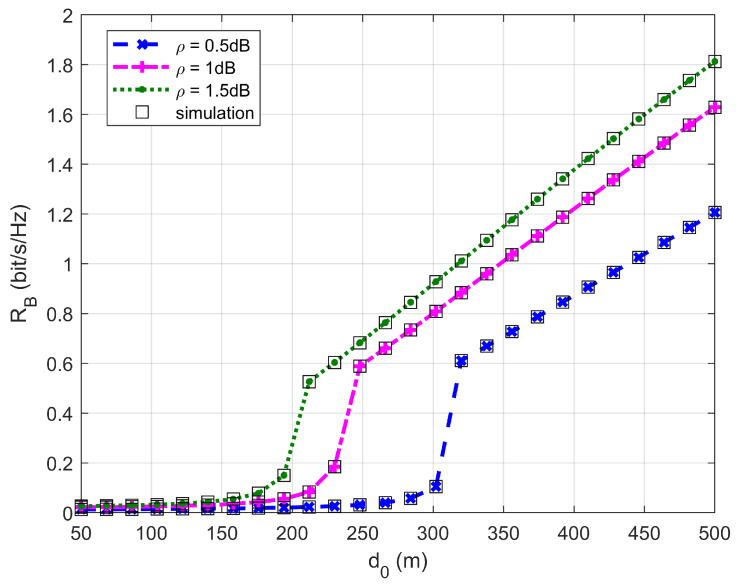
Covert rate versus $d_{0}$, where $d_{0}$ ranges from 50 to 500 m.

Figure 2, Figure 3, Figure 4 and Figure 5 plot the FWD’s flight altitude, the horizontal coordinate of the central position of D, the FWD’s transmit power, and the covert rate at which the FWD delivers data to Bob, where $d_{0}$ is between 15 and 50 m. Likewise, Figure 6, Figure 7, Figure 8 and Figure 9 show the simulation results when $d_{0}$ is between 50 and 500 m.

Figure 2 and Figure 6 show that the FWD’s flight altitude gradually decreases as $d_{0}$ increases. For a given $d_{0}$, the greater the uncertainty about noise power (namely, the larger the value of ρ), the lower the FWD’s flight altitude. When $d_{0}$ reaches approximately 20 m, a steep drop in the FWD’s flight altitude occurs. When $d_{0}$ is between 20 and 300 m, the FWD’s flight altitude decreases as $d_{0}$ increases. When $d_{0}$ is greater than 330 m, the FWD flies at the lowest altitude regardless of the value of ρ. These phenomena are consistent with the theoretical analysis, confirming the correctness of the investigations.

Figure 3 and Figure 7 show that the horizontal coordinate of the central position of D is on the line between Willie and Bob and satisfies the location restriction of D, confirming the correctness of the proposed algorithm.

Figure 4 and Figure 8 show that the FWD’s transmit power first decreases and then increases when $d_{0}$ ranges from 20 to 500 m. When $d_{0}$ is less than 20 m (in this situation $d_{0}\leq 4r_{R}^{*}$ holds), the FWD’s flight altitude is very high (see Figure 2) and hence, the FWD can operate at high transmit power levels. When $d_{0}$ reaches approximately 20 m, a steep drop in the FWD’s flight altitude occurs, resulting in a significant reduction in the FWD’s transmit power. When $d_{0}$ ranges between 20 and 200 m (in this situation $d_{0}>4r_{R}^{*}$ holds), the FWD can only operate at the minimum transmit power level. When $d_{0}$ is greater than 330 m, the FWD’s transmit power increases rapidly as $d_{0}$ increases. For a given $d_{0}$, the greater the uncertainty about noise power (namely, the larger the value of ρ), the higher the allowed transmit power.

Figure 5 and Figure 9 show that the covert rate increases as $d_{0}$ increases and the greater the noise uncertainty, the larger the achieved covert rate. When $d_{0}$ ranges from 15 to 200 m, the covert rate enhances slowly as $d_{0}$ increases. However, when $d_{0}$ is greater than 300 m, the covert rate enhances rapidly as $d_{0}$ increases.

In addition, Figure 2, Figure 3, Figure 4, Figure 5, Figure 6, Figure 7, Figure 8 and Figure 9 show that the theoretical results are consistent with the simulation results, confirming the correctness of the investigations.

**Figure 10:**
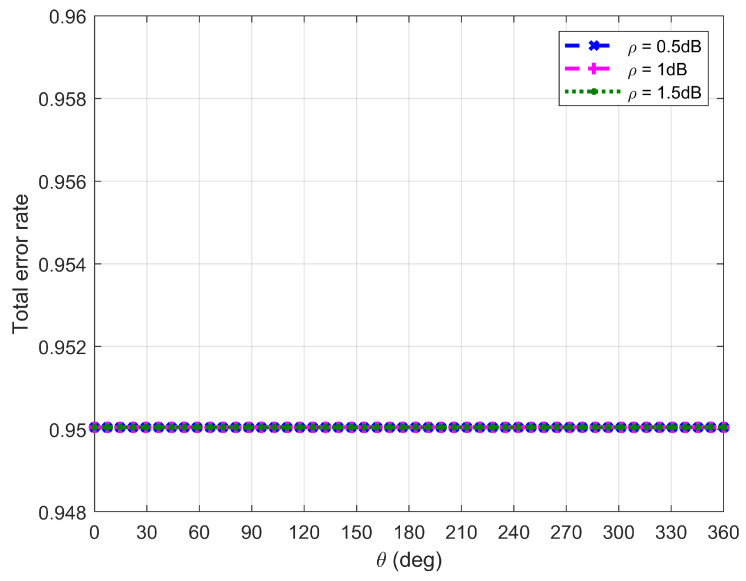
Willie’s DEP when $d_{0}$ is equal to 15 m.

**Figure 11:**
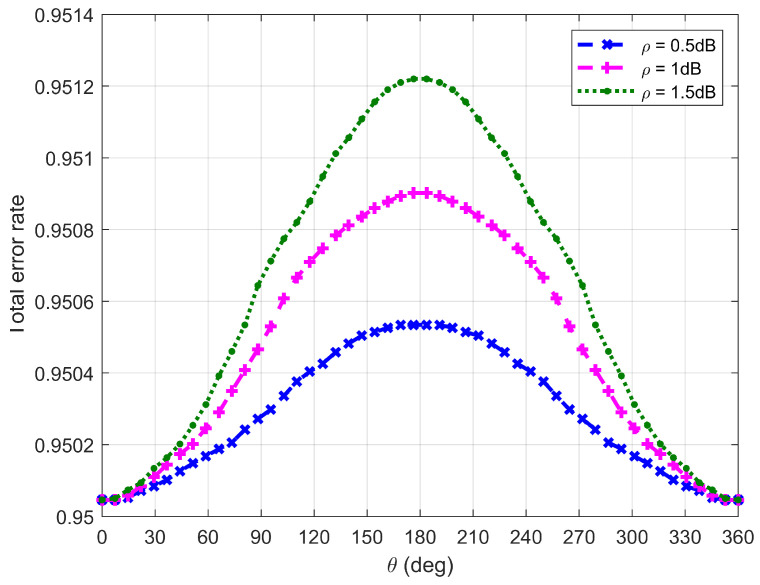
Willie’s DEP when $d_{0}$ is equal to 50 m.

**Figure 12:**
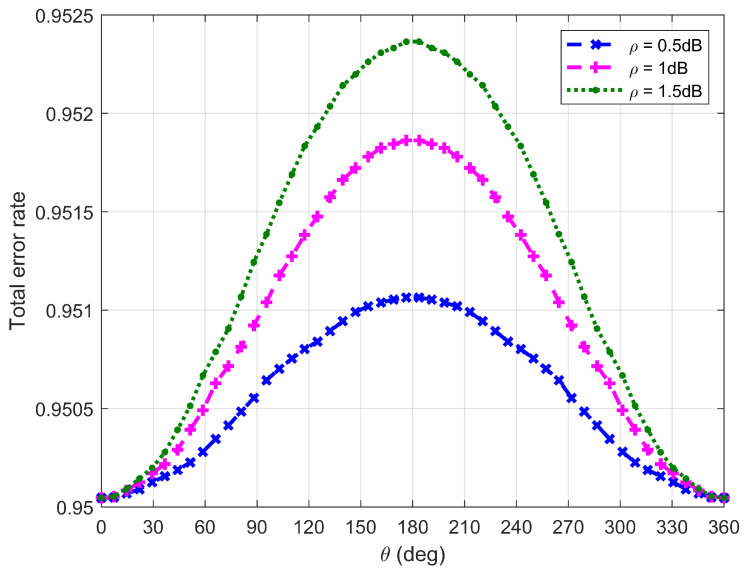
Willie’s DEP when $d_{0}$ is equal to 100 m.

**Figure 13:**
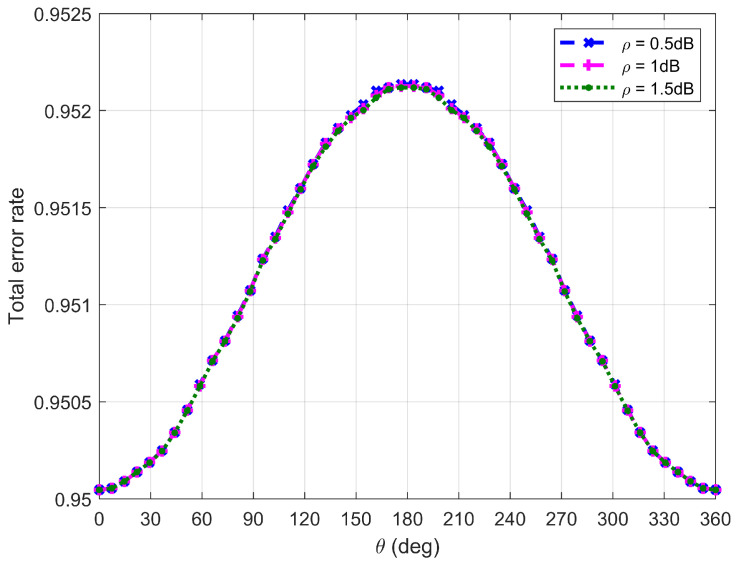
Willie’s DEP when $d_{0}$ is equal to 500 m.

Figure 10, Figure 11, Figure 12 and Figure 13 plot Willie’s DEP, where θ is the central angle when the FWD flies along the edge of the circular area D and is between $0^{\circ }$ and $360^{\circ }$. Figure 10, Figure 11, Figure 12 and Figure 13 correspond to four difference cases of $d_{0}=15\;m$, $d_{0}=50\;m$, $d_{0}=100\;m$ and $d_{0}=500\;m$, respectively.

It can be observed from Figure 10, Figure 11, Figure 12 and Figure 13 that Willie’s DEP is greater than the set value 1−ϑ, confirming the correctness of the proposed algorithm. For the cases where $d_{0}=50\;m$, $d_{0}=100\;m$ and $d_{0}=500\;m$, $\theta =180^{\circ }$ means that the FWD is close to Bob but far from Willie, which leads to a large DEP. For the case where $d_{0}=15\;m$, the DEP remains almost unchanged as θ varies. It is because the change in value of θ does not have any impact on the distance between the FWD and Willie if the FWD’s flight altitude is very high. Likewise, for the case where $d_{0}=15\;m$, noise uncertainty (i.e., the value of ρ) does not have any impact on Willie’s DEP since the FWD flies very high and the distance between the FWD and Willie is approximately invariant under changes in θ. For the cases where $d_{0}=50\;m$ and $d_{0}=500\;m$, however, Willie’s DEP enhances as the uncertainty about noise power (namely, increasing the value of ρ) increases. It is because a large value of ρ leads to great uncertainty about the noise power, making correct judgment more challenging for Willie. For the case where $d_{0}=500\;m$, the noise uncertainty (i.e., the value of ρ) will not affect Willie’s DEP as the FWD is far away from Willie.

Figure 14, Figure 15, Figure 16 and Figure 17 plot Bob’s outage probability when the FWD flies along the edge of D, where the four different cases considered in Figure 10, Figure 11, Figure 12 and Figure 13 are involved.

**Figure 14:**
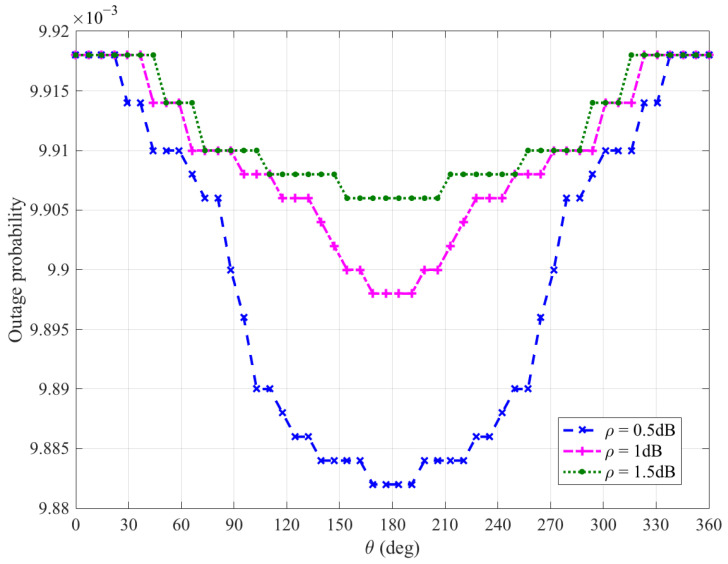
Bob’s outage probability when $d_{0}$ is equal to 15 m.

**Figure 15:**
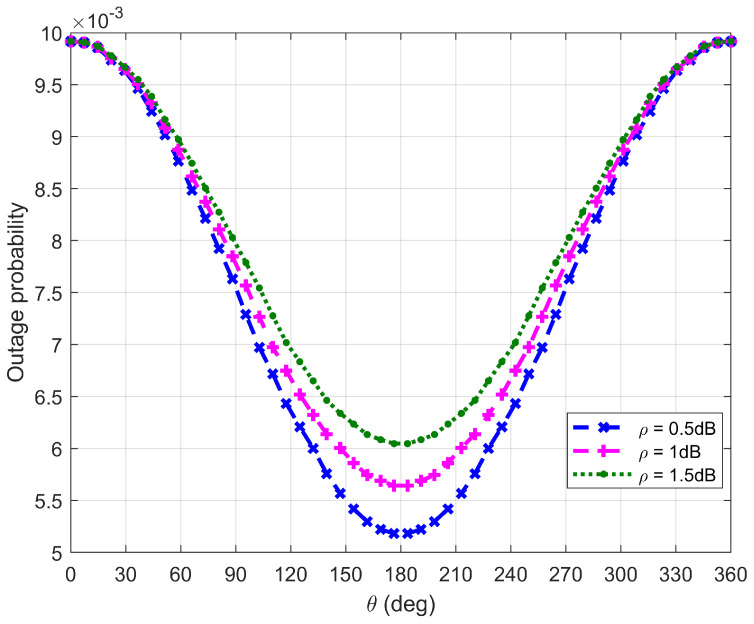
Bob’s outage probability when $d_{0}$ is equal to 50 m.

**Figure 16:**
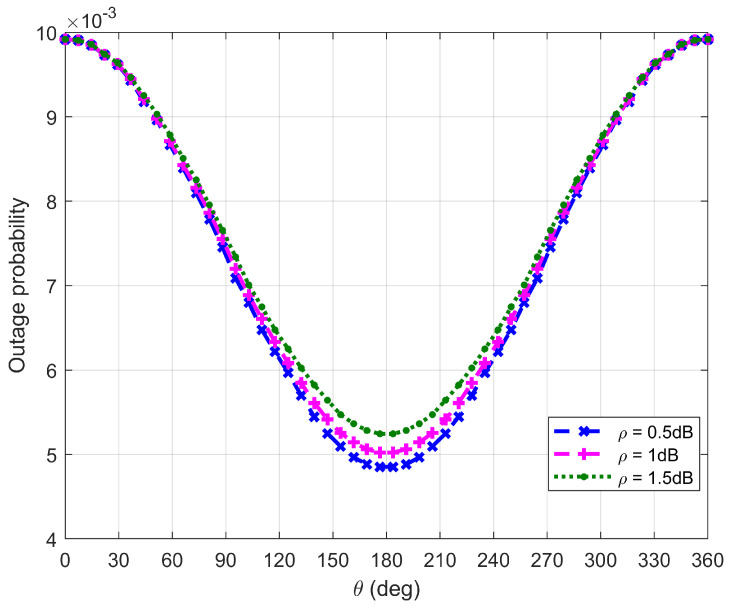
Bob’s outage probability when $d_{0}$ is equal to 100 m.

**Figure 17:**
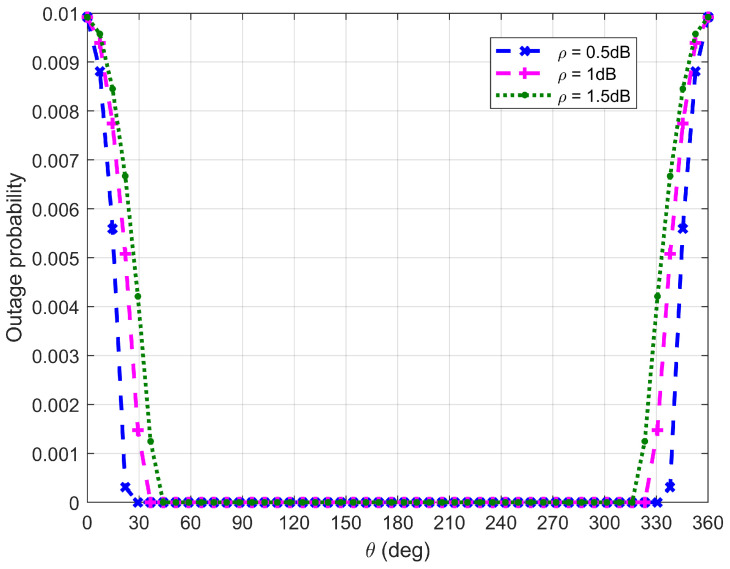
Bob’s outage probability when $d_{0}$ is equal to is 500 m.

It can be observed from Figure 14, Figure 15, Figure 16 and Figure 17 that Bob’s outage probability can be kept below the set value of 0.01, confirming the correctness of the proposed algorithm. When $\theta =180^{\circ }$, the FWD is close to Bob and then the minimum outage probability can be achieved. Figure 17 shows that when θ is between $45^{\circ }$ and $315^{\circ }$ (in this situation, the FWD is close to Bob), Bob’s outage probability tends to 0. Generally, Bob’s outage probability decreases as $d_{0}$ increases. It is because when $d_{0}$ is large, the FWD’s flight altitude is low and hence it can approach Bob, which is beneficial for data delivery. When noise uncertainty increases (namely, the value of ρ increases), Bob cannot accurately estimate the noise power, leading to SNR misestimation and consequently increasing the outage probability.

## 7. Discussion

An important finding is the threshold at $d_{0}=4r_{R}$. When $d_{0}>4r_{R}$, the receiver is far from the detector, lowering the altitude can improve the legitimate link more than increasing the detection risk, so the FWD should fly as low as possible. When $2r_{R}<d_{0}\leq 4r_{R}$, the receiver and detector are relatively close. Flying higher weakens the signal at both, but the reduction at the detector benefits covertness more. This explains why the FWD should fly as high as possible in this region.

Noise uncertainty improves covertness by increasing the detector’s DEP, thereby enabling higher transmit power or lower altitude. However, it also raises the receiver’s outage probability, which degrades the QoS. This trade-off indicates that noise uncertainty is not unconditionally beneficial. The results provide a quantitative guideline for selecting the operating point (altitude and power) given a specific level of noise uncertainty.

The findings offer several practical insights. First, if the distance between the receiver and detector can be estimated, the optimal altitude and transmit power can be determined using the proposed algorithm. Second, in environments with high noise uncertainty (e.g., dense urban areas), the system can tolerate lower altitudes or higher power to improve the covert rate, though the outage probability must be carefully considered. Third, the circular flight area respects the physical constraints of FWDs, making the solution practically deployable.

Several limitations remain in this article. First, the system model is idealized: it assumes an LoS channel, single static detector, and perfect knowledge of the receiver’s location. Second, only steady-state flight is considered; non-steady-state maneuvers (altitude changes) may temporarily affect covertness and QoS. Third, this article focuses on a single receiver; multi-user scenarios are not considered. Fourth, the detector’s location is assumed to be perfectly known; location uncertainty would require a more conservative strategy. Addressing these limitations is left for future work.

## 8. Conclusions

This article examined an FWD-enabled covert transmission, where the FWD performs an unrestricted level flight within a circular area with a radius of $r_{R}$ and meanwhile delivers covert data at a covert rate to a receiver situated $d_{0}$ meters away from a detector. With the aim of maximizing the covert rate while meeting the required QoS, a joint optimization algorithm is proposed, giving the optimal location and radius of the circular area and the optimal flight speed and transmit power of the FWD. It is found that the FWD should fly as high as possible if $2r_{R}<d_{0}\leq 4r_{R}$ and then deliver covert data with high transmit power. However, in this case, the covert rate remains relatively low. If $d_{0}>4r_{R}$, the FWD should fly as low as possible. In this situation, the FWD’s transmit power and the resulting covert rate are low if $d_{0}$ is slightly greater than $4r_{R}$. When $d_{0}$ increases, the FWD’s flight altitude decreases slowly, while progressively raising the transmit power. When $d_{0}$ increases to a certain value, the FWD flies at its minimum altitude. After that, the FWD’s transmit power and the covert rate increase rapidly as $d_{0}$ further increases. For a given $d_{0}$, the larger the uncertainty about noise power, the lower the allowed altitude or the higher the allowed transmit power, which increases the covert rate that can be achieved. In addition, the uncertainty about noise power is beneficial for increasing the detector’s DEP. However, it also leads to an increase in the receiver’s outage probability, declining the QoS level of the covert transmission.

## Figures and Tables

**Table 1:** Values of the parameters.

Parameter	Value	Parameter	Value
$P_{\min}$	0 dBm	$h_{\min}$	40 m
$P_{\max}$	23 dBm	$h_{\max}$	3000 m
$\beta_{0}$	−60 dB	$V_{\min}$	5 m/s
$\overset{⌣}{\delta }$	−120 dB	$V_{\max}$	50 m/s
ϑ	0.05	$\phi_{\max}$	$45^{\circ }$
$\Theta_{B}$	0.01	g	$9.8\;m/s^{2}$

## Data Availability

The original contributions presented in this study are included in the article. Further inquiries can be directed to the corresponding author.
